# Impact of Interstitial Pneumonia on the Survival and Risk Factors Analysis of Patients with Hematological Malignancy

**DOI:** 10.1155/2013/185362

**Published:** 2013-09-04

**Authors:** Wei-Liang Chen, Yu-Tzu Tsao, Tsun-Hou Chang, Tsu-Yi Chao, Woei-Yau Kao, Yeu Chin Chen, Ching-Liang Ho

**Affiliations:** ^1^Division of Hematology/Oncology, Department of Medicine, Tri-Service General Hospital, National Defense Medical Center, No. 325, Section 2, Cheng-Kung Road, Neihu, Taipei 114, Taiwan; ^2^Department of Medicine, Taoyuan General Hospital, No. 1492, Chung-Shan Road, Taoyuan City, Taoyuan County 330, Taiwan; ^3^Department of Radiology, Tri-Service General Hospital, National Defense Medical Center, No. 325, Section 2, Cheng-Kung Road, Neihu, Taipei 114, Taiwan

## Abstract

*Background*. The emergence of interstitial pneumonia (IP) in patients with hematological malignancy (HM) is becoming a challenging scenario in current practice. However, detailed characterization and investigation of outcomes and risk factors on survival have not been addressed. *Methods*. We conducted a retrospective study of 42,584 cancer patients covering the period between 1996 and 2008 using the institutional cancer registry system. Among 816 HM patients, 61 patients with IP were recognized. The clinical features, laboratory results, and histological types were studied to determine the impact of IP on survival and identify the profile of prognostic factors. *Results*. HM patients with IP showed a significant worse survival than those without IP in the 5-year overall survival (*P* = 0.027). The overall survival showed no significant difference between infectious pneumonia and noninfectious interstitial pneumonia (IIP versus nIIP) (*P* = 0.323). In a multivariate Cox regression model, leukocyte and platelet count were associated with increased risk of death. *Conclusions*. The occurrence of IP in HM patients is associated with increased mortality. Of interest, nIIP is a prognostic indicator in patients with lymphoma but not in patients with leukemia. However, aggressive management of IP in patients with HM is strongly advised, and further prospective survey is warranted.

## 1. Introduction

Pulmonary disorder is frequently encountered in patients with hematological malignancy (HM), and it occasionally acts as the treatment-associated complication during the course of HM [1]. The occurrence of pulmonary complications in HM patients undergoing stem cell transplantation or chemotherapy had been reported to carry high risks of morbidity and mortality [2, 3]. Several conditions of pulmonary events had been described, such as thromboembolism, hemorrhage, inflammation, fibrosis, and infection, but their clinical impact on prognosis had rarely been studied. Of importance, interstitial pneumonia (IP) in HM patients is becoming a challenging scenario in current practice because of its protean, multifaceted nature. IP is a heterogeneous group of diseases characterized by diffuse parenchymal lung disorders that can be classified into noninfectious interstitial pneumonia (nIIP) and infectious interstitial pneumonia (IIP) according to distinct clinical, radiological, and histopathological features [4]. The IIP and nIIP had been implicated with a worse outcome in few anecdotal reports but detailed characterization and investigation of outcomes and risk factors on survival have not been addressed. The aim of this study was to determine the impact of IP on survival and identify the profile of prognostic factors.

## 2. Materials and Methods

**Figure 1:**
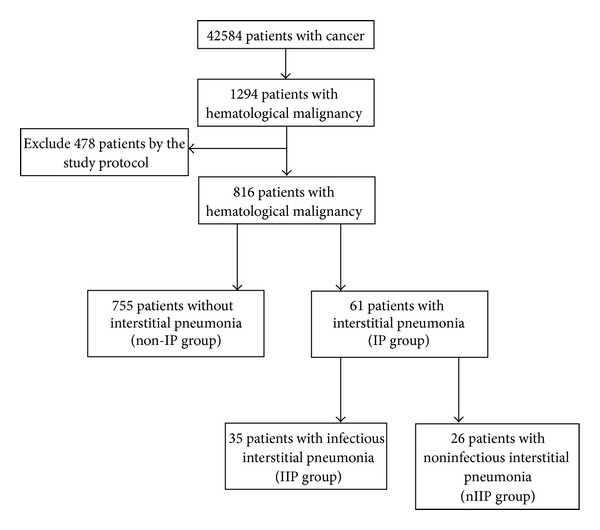
Study design in patients with hematological malignancy during the study period. (non-IP group: the HM patients without IP; IP: interstitial pneumonia; IIP: infectious interstitial pneumonia; nIIP: noninfectious interstitial pneumonia).

This retrospective study was conducted at Tri-Service General Hospital (TSGH). It was approved by the Institutional Review Board of TSGH in accordance with the revised Helsinki Declaration. We searched the TSGH database of cancer registries for cases between 1996 and 2008 with a diagnosis of HM. Based on follow-up data from the cancer registry system, a total of 42584 cancer patients were recorded, and 1294 patients with HM were identified. To focus on the association between HM and IP more precisely, we excluded individuals with human immunodeficiency virus infection, heart failure, connective tissue disease, sarcoidosis, and underlying coexisting solid tumors. In total, 816 patients with HM were eligible and enrolled in this study (Figure 1). There were 170 cases of acute myeloid leukemia (ALL), 90 cases of acute lymphoid leukemia (AML), 87 cases of chronic myeloid leukemia (CML), 26 cases of chronic lymphoid leukemia (CLL), 30 cases of Hodgkin's lymphoma (HL), 304 cases of non-Hodgkin's lymphoma (NHL), and 109 cases of multiple myeloma (MM).

The comprehensive chart reviews of patients with HM were done by a panel of five experienced doctors. 61 patients carried a diagnosis of IP (including IIP and nIIP), based on medical history, physical examination, and abnormalities compatible with bilateral lung disease on high resolution computed tomography (honeycombing with basal and peripheral predominance, peripheral reticular shadow, or ground-glass opacity) and constituted the study group. The diagnosis of IIP was confirmed based on the identification of pathogen in sputum or bronchoalveolar lavage fluid (recognized through series of cultures, polymerase chain reaction, or histological identification from transthoracic needle biopsy, transbronchial biopsy, video-assisted thoracoscopic surgery, or open lung biopsy). The histopathological diagnosis of nIIP was verified by surgical open lung biopsy in 16 patients and transbronchoscopic lung biopsy in 10 patients. The types of nIIP were classified according to recently reclassified American Thoracic Society/European Respiratory Society criteria [4]. All cases of nIIP showed no evidence of concurrent pulmonary infection. 

All clinical information including demographic findings, clinical presentations, physical examinations, histopathological reports, radiological features, and laboratory results were investigated. All patients in the study were followed up by medical record review until death, last visit at our institute.

### 2.1. Statistical Analysis

All the statistical analyses were performed using the Statistical Package for the Social Sciences (SPSS version 14.0 for Windows, SPSS, Chicago). Two-sided *P* values of less than 0.05 were considered to indicate significance. Quantitative parameters were expressed as mean and standard deviation, while qualitative data were presented as number and percentage. The patients' characteristics were compared using the chi-square test for discrete variables and the independent *t*-test or Wilcoxon Rank sum test for continuous variables. Survival rates were calculated from the time of initial diagnosis to the date of last follow-up or death. Kaplan-Meier survival curves were plotted to ascertain the relationship of the various types of HM with IP and subsequent mortality. Univariate comparisons were made using the log rank test. In a multivariate analysis of survival, the Cox regression model was used to determine the effects of different variables on overall survival.

## 3. Results

### 3.1. Characteristics of HM Patients with and without IP

Among the 816 patients with HM, 61 patients with IP were identified (Table 1). Of the 61 patients, 41 were males and 20 were females with a mean age of 50.3 years (range, 7–87 years). In the IP group, 26 (42.6%) patients had NHL, 5 (8.2%) had HL, 10 (16.4%) had ALL, 8 (13.1%) had AML, 7 (11.5%) had CML, and 5 (8.2%) had MM. In the non-IP group, NHL was the most common HM (*n* = 278, 36.8%). 61 HM patients with either IIP or nIIP constituted the study group (IP group). Among 26 patients classified as nIIP, there were 25 patients diagnosed with nonspecific interstitial pneumonia (NSIP) and one patient diagnosed with bronchiolitis obliterans organizing pneumonia (BOOP). In the remaining 35 patients with diagnosis of IIP, 28 patients had only the pneumocystis jiroveci pneumonia (PJP) infection. Five patients were diagnosed with coinfection with three pathogens: PJP, herpes simplex virus (HSV), and cytomegalovirus (CMV). Two patients were coinfected with two pathogens: PJP and CMV.

### 3.2. Survival of IP and Non-IP Groups in Patients with HM

**Figure 2:**
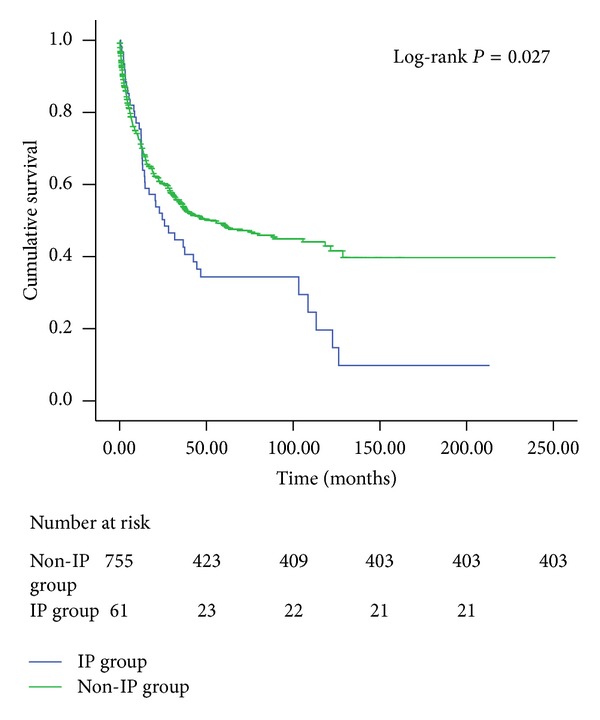
Kaplan-Meier survival probability curve of 816 patients with HM according to the IP group and the non-IP group.

The median survival duration of non-IP group (*n* = 755) was 36.9 months (range, 0.3–249.5 months). After 12 years of follow-up, 46.6 percent of patients (*n* = 352) had died. In the IP group of 61 patients, follow-up was completed to death for 40 patients (65.6%), and mean follow-up for living patients was 38.6 months (range, 0.6–213.2 months). 5-year overall survival was significantly lower for the IP group than for the non-IP group (*P* = 0.027) (Figure 2). The major cause of death in the three groups was shown in Table 2. In the non-IP group, sepsis/multiple organ dysfunction syndrome represented the most common cause of death (45%), followed by disease progression (25%) and pulmonary events (8%). In the IP group, the pulmonary causes were the second leading cause of death.

### 3.3. Survival Analysis and Risk Factors of Death of HM Patients with IP

Univariate analysis illustrated that age, types of HM, hemoglobin, platelet count, and arterial carbon dioxide partial pressure were statistically significant for risk of mortality (Table 3). The Cox's proportional hazard model including all recorded variables disclosed leukocyte and platelet count to be the independent predictors of survival, while the others failed to achieve significance in multivariate testing (Table 4). Moreover, there was a positive trend between the hemoglobin levels and long-term survival (*P* = 0.051).

### 3.4. Survival of Patients with IP in Different HM

**Figure 3:**
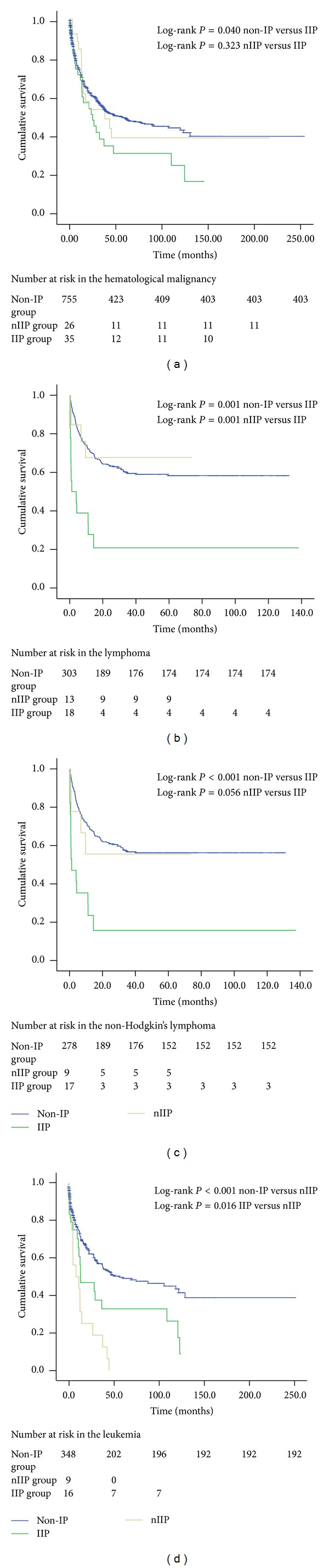
Overall survival of patients with IIP and nIIP groups in different HM. (a) Survival of HM patients comparing IIP and nIIP groups. (b) Survival of patients with lymphoma comparing IIP and nIIP groups. (c) Survival of NHL patients comparing IIP and nIIP groups. (d) Survival of leukemia patients comparing IIP and nIIP groups.

In all patients with HM, there was no significant difference between nIIP and IIP groups during the follow-up period (*P* = 0.323) (Figure 3(a)). Survival rates were significantly higher in the non-IP group than in the IIP group (*P* = 0.040) (Figure 3(a)). For lymphoma, the nIIP group had a significantly better survival than the IIP group (*P* = 0.001) (Figure 3(b)). For NHL, Kaplan-Meier survival analysis demonstrated the non-IP group had a significantly better survival than the IIP group (*P* < 0.001) (Figure 3(c)). In contrast, survival analysis of 25 leukemia patients showed that nIIP patients had worse survival than IIP patients (*P* = 0.016) (Figure 3(d)). 

### 3.5. IIP versus nIIP in the Patients with HM

**Figure 4:**
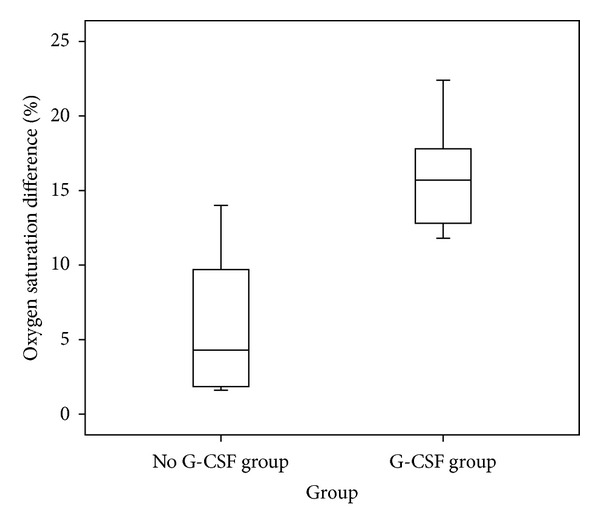
Difference of oxygen saturation in IP patients with treated G-CSF and without using G-CSF.

The characteristics of the patients stratified into IIP and nIIP groups are shown in Table 5. The mean age of the 35 patients with IIP was 46.9 years (range 7–87 years). There were no significant differences between the IIP and nIIP groups with respect to age and gender. The time between the last treatment and the development of IP was shorter in the patients with IIP than in those with nIIP. There were no differences between the two groups in the laboratory data during the development of IP, except serum C-reactive protein (CRP) which was higher in the IIP group than in the nIIP group. There were increased levels of serum lactate dehydrogenase in 88.6% of the IIP group and 61.5% of nIIP group. Notably, the serum lactate dehydrogenase levels in the IIP group were significantly higher than those in the nIIP group (*P* = 0.029). Of 61 patients with IP, 14 patients with nIIP and 6 patients with IIP had received the granulocyte colony-stimulating factor (G-CSF) treatment before the emergence of IP. The difference of oxygen saturation obtained from the pulse oximetry between the time of pathologically proven HM and the time of onset of IP was shown in Figure 4. There was no significant difference in the oxygen saturation at baseline. The difference in oxygen saturation in the IP patients who were treated with G-CSF was higher than in those who did not receive G-CSF (15.6 ± 3.2, 5.8 ± 4.5, *P* < 0.001).

### 3.6. The Treatments of IIP and nIIP in the Patients with HM

**Figure 5:**
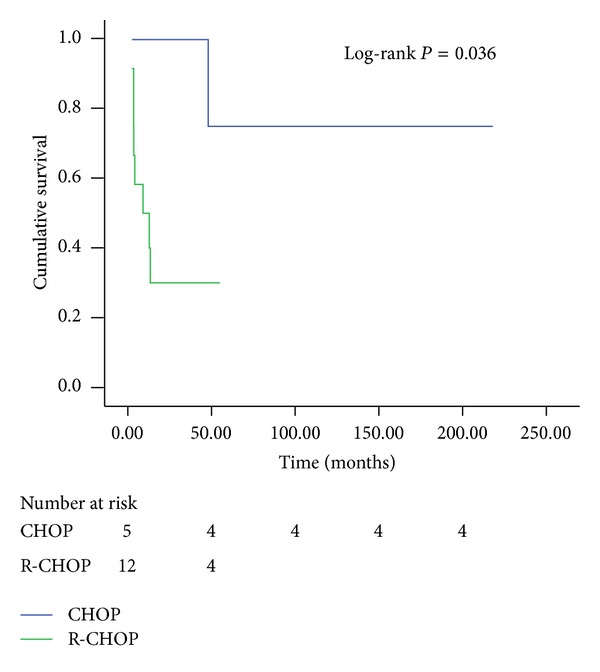
Kaplan-Meier survival probability curve of 17 patients with DLBCL comparing R-CHOP regimen and CHOP regimen.

In the nIIP group, six of 25 patients with NSIP and one patient with BOOP developed chemotherapy-related IP (Table 6). For the treatment of patients with nIIP, three-day course of intravenous methylprednisolone achieved clinical improvement (22 NSIP patients and 1 BOOP patient). Three patients were unresponsive to the treatment and died of respiratory failure. Among the 35 patients with IIP, none received PJP prophylactic regimens or antiviral prophylaxis. In contrast, all of the patients with PJP received treatment with trimethoprim-sulfamethoxazole within 48 hours of the onset of symptoms. The median duration of therapy was 14 days (range 1–45 days). The clinical presentations in the most patients improved after treatment. During antipneumocystis medication, the concurrent use of corticosteroids (methylprednisolone, 50–80 mg/d for an average of 13 days) was administered to 11 patients (31.4%). Five patients with CMV infection received course of intravenous ganciclovir (5 mg/kg twice daily) with satisfactory response. Considering treatment-induced IP, we investigated 17 patients with diffuse large B-cell lymphoma (DLBCL) in 26 patients with NHL. Of these 17 patients, 8 had nIIP and 9 had IIP. The mean survival of these 17 patients was 34.6 months (range 2.2–213.2 months). Twelve of the 17 (70.6%) patients with DLBCL received the R-CHOP regimen (rituximab, cyclophosphamide, epirubicin, vincristine, and prednisone), and the remaining 5 patients received the CHOP regimen (cyclophosphamide, epirubicin, vincristine, and prednisone). For DLBCL patients with coexisting IP, the overall survival curves of the patients treated with R-CHOP demonstrated a worse outcome than those treated with CHOP (*P* = 0.036, Figure 5).

### 3.7. The Carbon Monoxide Diffusing Capacity of the HM Patients with nIIP

**Figure 6:**
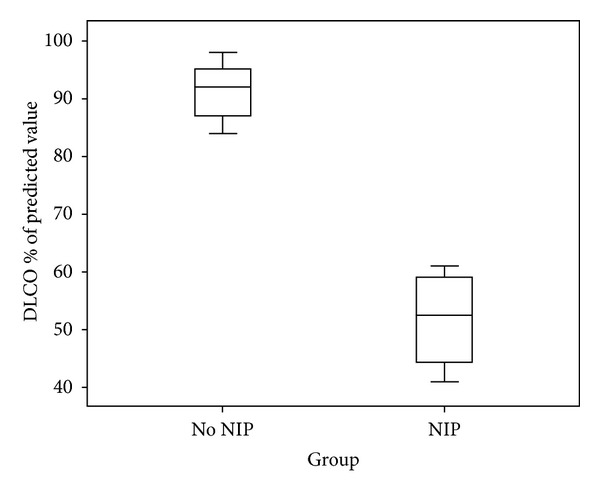
DLCO in HM patients with and without nIIP.

For the long-term sequelae of lung function in the nIIP group (Figure 6), 4 out of 26 patients with nIIP underwent evaluation of carbon monoxide diffusing capacity (DLCO) at mean 2.7 months follow-up after recovery from nIIP. The estimation of DLCO was not available in IIP patients. In addition, we studied the DLCO in 15 HM patients without IP (the control group). Compared with the control group, the follow-up DLCO level in the nIIP group was significantly worse (*P* < 0.001).

## 4. Discussion 

Compared with non-IP group, the second dip in the IP group (Figure 2) showed that there were increased numbers of death around the 100 months. The long-term impact of IP may produce irreversible vascular and alveolar damage, abnormal gas exchange, fibrosis, declined pulmonary function, and cardiopulmonary compromise. We believed that the hematological malignancy and anticancer modalities may insult cardiopulmonary function and immune systems, and ensuing interstitial pneumonia can result in physiological restriction and immune system dysregulation that harboured a predisposing milieu for sepsis and respiratory failure.

IP of infectious origin, particularly PJP, in patients with HM poses diagnostic challenges. In previous studies, the therapeutic strategy and clinical characteristics of IP secondary to PJP in patients treated with immunosuppressive therapy or those with AIDS have been emphasized [5–7]. In the current study, NHL was the most frequent HM in the patients who developed PJP during treatment. Interestingly, PJP was not observed in the patients with CLL, in contrast to data reported by Sepkowitz [5]. The discrepancy between these investigations may be a result of the lower incidence of CLL in Asian than in Western countries [8]. In the majority of cases, PJP (25 episodes, 40.9%) was documented in the patients who were in the induction/reinduction phases of chemotherapy. 

Progressive nIIP is a life-threatening complication during treatment for HM. In our study, the most common underlying malignancy was NHL, accounting for 36% of patients (*n* = 9) with proven nIIP. Nakase et al. analysed 14 HM patients with acute interstitial pneumonitis during chemotherapy, of which the majority (eight patients, 57.1%) were diagnosed with NHL [8]. To date, the lack of a clear understanding of the aetiology of nIIP remains one of the main stumbling blocks in establishing its association with HM. The fact that nIIP tends to occur during the recovery phase of chemotherapy-induced leukopenia has been attributed to a wide variety of cytokines, including G-CSF or granulocyte-macrophage colony-stimulating factor (GM-CSF) [9, 10], since G-CSF- or GM-CSF-related lung injuries have been reported [11, 12]. G-CSF increases the number of neutrophils coupled with enhancement of neutrophil function, and these neutrophils are prone to be trapped by pulmonary vascular capillaries and to release oxygen radicals and proteolytic enzymes [13]. Of 61 HM patients with IP, 20 patients (32.8%) received G-CSF, 12 (60%) of whom received this treatment during hematopoietic recovery phase of induction/re-induction chemotherapy. Consistent with previous studies, our findings disclosed the possibility that dynamic recovery of neutrophils in HM patients receiving intensive chemotherapy may be associated with increased risk of IP.

Our retrospective study revealed that those DLBCL patients treated with the CHOP regimen who developed IP appeared to have a more favorable prognosis than those treated with R-CHOP, with an estimated five-year survival rate of 80%. Studies have shown that, for DLBCL, the R-CHOP regimen has better therapeutic outcomes than the CHOP regimen in terms of response rate and outcome survival [14, 15]. However, in the life-threatening event of IP, the outcomes may be the reversed. Rituximab targets the CD20 cell surface protein located on mature B-cells and most B cell malignancies. Its mechanisms of action include complement-dependent cell lysis, cell-mediated cytotoxicity, and induction of apoptosis [16, 17]. Most adverse events result from an infusion-related symptom complex; however, severe pulmonary complications are rare. Liu et al. reported 9 patients with NHL in whom interstitial pneumonitis developed after rituximab-containing chemotherapy [18]. Tonelli et al. reported a case of hypersensitivity pneumonitis with classic radiographic and histopathological findings after rituximab treatment [19]. Similarly, treatment with rituximab with or without CHOP resulted in pulmonary deterioration in two reported cases, one of which was fatal [20, 21]. The mechanisms of rituximab-induced interstitial pneumonitis remain unclear. However, dysregulated cellular cytotoxicity may be related to a mechanism of delayed pulmonary toxicity [22]. As a result, it is tempting to speculate that the poor results of the R-CHOP group in our study were attributable to increased severe pulmonary complications related to rituximab.

It is becoming increasingly evident that physiological parameters, such as DLCO, are useful in predicting survival and identifying the disease severity [23, 24]. In our study, the follow-up DLCO level in the IP group was significantly worse than that in the non-IP group. The interpretation of the results highlighted that the measure of DLCO is imperative in HM patients with IP for the surveillance of pulmonary function. It might be more interesting to consider evolution of DLCO before and after diagnosis of nIIP. However, the longitudinal change of DLCO during the disease course of nIIP was not delineated because of our retrospective design. A more sophisticated study design is needed to explore the associations between the DLCO and nIIP.

However, there were some limitations to our study. During a 21-year study period, we identified all patients with HM who developed IP. The number of cases described in the series is small and retrospective. However, to the best of our knowledge, this survey is one of only a few to elucidate the long-term influence of IP in patients with HM [25, 26]. The exact nature of the interactions between IP and HM remains unclear. Comparisons of the impacts of IP on patients with HM were difficult because of the heterogeneity of the patient populations as well as the multiplicity of factors that ultimately determined their survival. In addition, different outcomes are likely to be related to the use of various therapeutics for HM.

In conclusion, the occurrence of IP in HM patients is associated with increased mortality. Of interest, nIIP is a prognostic indicator in patients with lymphoma but not in patients with leukemia. The decline of follow-up DLCO level in the nIIP group was observed during the study period. Recognizing the distinct manifestations of IIP and nIIP in the different type of HM has allowed a better understanding of the disorders. However, aggressive management of IP in patients with HM is strongly advised, and further prospective survey is warranted.

## Figures and Tables

**Table 1:** Demographics of HM patients with and without IP.

Patients	IP group			Non-IP group
	IIP	nIIP	Total	
Sex (male/female)	23/12	499/256	41/20	499/256
Mean age (years; range)	46.9 (7–87)	52.1 (2–93)	50.3 (7–87)	52.1 (2–93)
Median follow-up period (months)	8.7	26.7	8.7	26.7
Underlying hematological disease				
Non-Hodgkin's lymphoma	**17**	**278 (36.8%)**	**26 (42.6%)**	**278 (36.8%)**
B-cell NHL	10	223	19	223
T-cell NHL	7	55	7	55
Hodgkin's lymphoma	**1**	**25 (3.3%)**	**5 (8.2%)**	**25 (3.3%)**
Acute lymphoblastic leukemia	**8**	**80 (10.6%)**	**10 (16.4%)**	**80 (10.6%)**
Pre-B ALL	5	59	6	59
T-cell ALL	2	17	3	17
B-cell ALL	1	4	1	4
Acute myeloid leukaemia	**5**	**162 (21.5%)**	**8 (13.1%)**	**162 (21.5%)**
Chronic myeloid leukaemia	**3**	**80 (10.6%)**	**7 (11.5%)**	**80 (10.6%)**
Chronic phase	0	—	0	—
Accelerated phase	1	—	5	—
Blast crisis	2	—	2	—
Chronic lymphoblastic leukaemia	**0**	**26 (3.4%)**	**0**	**26 (3.4%)**
Multiple myeloma	**1**	**104 (13.8%)**	**5 (8.2%)**	**104 (13.8%)**
Total	**35 (57%)**	**755**	**61**	**755**

HM: hematological malignancy; IP: interstitial pneumonia; IIP: infectious interstitial pneumonia; nIIP: noninfectious interstitial pneumonia; non-IP group: the hematological malignancy patients without interstitial pneumonia; NHL: non-Hodgkin's lymphoma; ALL: acute lymphoblastic leukaemia.

**Table 2:** Distribution of major causes of death in different groups.

Group∖cause of death	IP group		Non-IP group
	IIP group	nIIP group	
Sepsis/MODS	10 (40%)	6 (40%)	157 (45%)
Progressive disease	5 (20%)	3 (20%)	89 (25%)
Pulmonary causes	6 (24%)	4 (26%)	28 (8%)
Neurologic causes	0 (0%)	0 (0%)	17 (5%)
Cardiac causes	1 (4%)	0 (0%)	11 (3%)
Renal causes	1 (4%)	1 (7%)	25 (7%)
Hemorrhage	1 (4%)	1 (7%)	11 (3%)
Others	1 (4%)	0 (0%)	14 (4%)

Total	25 (100%)	15 (100%)	352 (100%)

IP group: hematological malignancy patients with interstitial pneumonia; non-IP group: hematological malignancy patients without interstitial pneumonia; MODS: multiple organ dysfunction syndrome; IIP: infectious interstitial pneumonia; nIIP: non-infectious interstitial pneumonia.

**Table 3:** Univariate comparisons between IP patients with and without death.

Variables	Alive group (*n* = 21)	Death group (*N* = 40)	*P* value
Male sex	16	25	0.391
Age	40.0 ± 24.9	55.7 ± 24.8	0.023
Underlying hematological malignancy			0.012
Non-Hodgkin lymphoma	8	18	
Hodgkin lymphoma	5	0	
Leukemia	6	19	
Multiple myeloma	2	3	
Hemoglobin (g/dL)	11.6 ± 2.4	9.6 ± 1.7	0.001
Leukocyte count(/uL)	6210 ± 3600	10948 ± 10671	0.540
Platelet count (×10^3^/uL)	222476 ± 111744	124050 ± 114500	0.002
C-reactive protein (mg/dL)	7.94 ± 9.5	9.6 ± 1.7	0.311
Arterial pO_2_ (mmHg)	83.6 ± 12.9	78.9 ± 11.8	0.169
Arterial pCO_2_ (mmHg)	36.7 ± 4.1	34.2 ± 4.1	0.026

IP: interstitial pneumonia.

**Table 4:** Predictor of death by multivariate Cox regression analysis applied to HM patients with IP.

Variables	HR (95% CI)	*P* value
Male sex	1.697 (0.314–9.180)	0.539
Age	1.031 (0.997–1.065)	0.070
Hemoglobin (g/dL)	0.628 (0.394–1.003)	0.051
Leukocyte count (/uL)	1.001 (1.001–1.003)	0.012
Platelet count (×10^3^/uL)	0.999 (0.998-0.999)	0.002
C-reactive protein (mg/dL)	1.033 (0.931–1.146)	0.540
Arterial pO_2_ (mmHg)	0.985 (0.909–1.046)	0.479
Arterial pCO_2_ (mmHg)	0.927 (0.731–1.175)	0.532

**Table 5:** Clinical and laboratory features of IP group.

Measured parameter	IP group		*P* value
	IIP group	nIIP group	
Sex (males/females)	23/12	18/8	0.989
Age (years)	46.9 (7–87)	54.6 (14–86)	0.204
Smoker	7	5	0.940
COPD	3	2	0.901
Asthma	1	1	1.000
Time from the treatment of HM to IP (mean, months)	1.9 (0.1–17.7)	4.1 (0.1–31.5)	0.017
Episodes of IP	1.0 ± 0	1.2 ± 0.4	0.022
Hemoglobin (g/dL)	9.9 ± 1.93	10.8 ± 2.47	0.177
Leukocyte count (/uL)	9734 ± 11050	8756 ± 5809	0.258
Neutrophils percentage (%)	64.6 ± 25.16	66.3 ± 20.06	0.948
Lymphocyte percentage (%)	15.9 ± 12.89	17.3 ± 10.78	0.366
Platelet count (×10^3^/uL)	146.2 ± 122.64	173.7 ± 121.95	0.347
C-reactive protein (mg/dL)	11.7 ± 7.35	5.9 ± 5.67	0.002
LDH (%) (>250 U/L)	88.6%	61.5%	0.029
Use of G-CSF	17.1% (6/35)	54.8% (14/26)	0.020
Arterial pO_2_ (mmHg)	79.21 ± 12.51	82.4 ± 12.10	0.507
Arterial pCO_2_ (mmHg)	34.4 ± 4.06	36.0 ± 4.36	0.137
Stem cell transplantation	2	3	0.642
Radiotherapy	2	4	0.387

IIP: infectious interstitial pneumonia; nIIP: noninfectious interstitial pneumonia; COPD: chronic obstructive pulmonary disease; HM: hematological malignancy; IP: interstitial pneumonias; LDH: lactate dehydrogenase.

**Table 6:** Reviews of patients with HM and chemotherapy-related IP.

Case	Age/sex	Hematological disease	Presenting symptoms	Chemotherapy	Number of cycles	Clinicopathological diagnosis	Outcome
1	M/42	DLBCL	Cough, dyspnea	R-CHOP	2	NSIP	Recovery
2	F/23	ALL	Fever	Induction chemotherapy	1	NSIP	Recovery
3	M/55	DLBCL	Cough, fever	R-CHOP	1	NSIP	Died
4	M/62	DLBCL	Fever, dyspnea	CHOP	1	NSIP	Recovery
5	M/55	DLBCL	Fever	R-CHOP	2	NSIP	Died
6	M/60	AML	Cough	Induction chemotherapy	1	NSIP	Died
7	M/17	Hodgkin's lymphoma	Fever, dyspnea	ABVD	6	BOOP	Recovery

DLBCL: diffuse large B-cell lymphoma; ALL: acute lymphoblastic leukaemia; AML: acute myeloid leukaemia; R-CHOP: rituximab, cyclophosphamide, epirubicin, vincristine, and prednisone; CHOP: cyclophosphamide, epirubicin, vincristine, and prednisone; ABVD: doxorubicin, bleomycin, vinblastine, and dacarbazine; NSIP: nonspecific interstitial pneumonia; BOOP: bronchiolitis obliterans organizing pneumonia.
